# Deregulation and pricing of medical services: a policy experiment based in China

**DOI:** 10.1186/s12913-021-06525-x

**Published:** 2021-05-25

**Authors:** Wenbin Zang, Mei Zhou, Shaoyang Zhao

**Affiliations:** 1 School of Insurance, Southwestern University of Finance and Economics, Chengdu, China; 2 School of Public Administration, Southwestern University of Finance and Economics, Chengdu, China; 3 School of Economics, Sichuan University, Chengdu, China

**Keywords:** Price regulation, Private hospitals, Information advantage, Medical expenditure

## Abstract

**Background:**

Price regulation is a common constraint in Chinese hospitals. Based on a policy experiment conducted in China on the price deregulation of private nonprofit hospitals, this study empirically examines the impact of medical service price regulation on the pricing of medical services by hospitals.

**Methods:**

Using the claim data of insured inpatients residing in a major Chinese city for the period 2010–2015, this study constructs a DID (difference-in-differences) model to compare the impact of price deregulation on medical expenditure and expenditure structure between public and private nonprofit hospitals.

**Results:**

The empirical results based on micro data reveal that, price deregulated significantly increased the total expenditure per inpatient visit by 10.5%. In the itemized expenditure, the diagnostic test and drug expenditure per inpatient visit of private nonprofit hospitals decreased significantly, whereas the physician service expenditure per inpatient visit increased significantly. For expenditure structure, the proportions of drug expenditure and diagnostic test expenditure per inpatient visit significantly decreased by 5.7 and 3.1%, respectively. Furthermore, this paper also found that hospitals had larger price changes for dominant diseases than for non-dominant diseases.

**Conclusions:**

Under price regulation, medical service prices generally become lower than their costs. Therefore, after price deregulation, private nonprofit hospitals increase medical service prices above their cost and achieve the service premium increasing physician medical services. Further, although price deregulation causes patient expenditure to increase to a certain level, it optimizes the expenditure structure, as well.

## Background

In China, the majority of hospitals, particularly public hospitals, have to adhere to very strict price regulations. Hospitals must follow government-specified price regulation standards while pricing their medical services and drugs. The purpose of price regulation is to maintain fairness in access to medical services by reducing the price of basic medical services, usually below the marginal cost [1]. In compensation, subsidies and tax incentives are provided by the government, and barriers to entry in the medical market are also backed by the government. However, government subsidies to public hospitals have been gradually decreased after the medical marketization reform, accounting for less than 10% of the medical revenue of hospitals. Public hospitals have to set high prices for other services to maintain a reasonable level of revenue, especially new drugs and high-tech diagnostic test, such as CT and MRI scans [1, 2]. Therefore, the distorted incentive mechanism caused by price regulation may undermine the efficiency and fairness of medical system. Price regulation has long been criticized for excessive medical treatment in China [2–4].

Price regulation distorts the signaling role played by price in resource allocation. Due to the presence of relatively serious information asymmetry in the medical service market, price regulation will lead to excessive medical treatment. Since Pricing and payment is predominantly on a FFS (fee-for-service) basis, hospitals and doctors tend to avoid the price regulation of medical services by drug overusing and diagnostic over-testing [1, 2, 5]. For example, after the implementation of the Zero Markup Drug Policy, hospitals and pharmaceutical companies conspired to increase drug prices and deny the market to low-price drugs. When the government controls the drug revenue proportion, hospitals compensate for this decline in proportion by increasing their diagnostic test expenditure [6–10].

When the prices of most of the basic medical services and drugs are regulated, hospitals achieve their profits mainly by inducing demand and doctors play a significant role in this process of inducing demand [11–18]. As multitasking agents, doctors are often simultaneously entrusted various tasks by both hospitals and patients. As the patients’ agent, a doctor’s task is to provide the patients with professional medical services and maximize their benefit in the diagnosis process. Further, as the hospital’s agent, a doctor must not only provide diagnoses for patients but also improve income for the hospital and satisfy other assessment indicators of the hospital. However, while acting as agents, doctors may feel conflicted when their personal interests contradict patients’ interests [19–23]. For example, Lu [20] pointed out that when offered economic incentives, doctors might deviate from their agent obligations and use information advantages to induce patients with a strong ability to pay for more medical services to maximize their personal interest. On encountering price regulation, such as a reduction in the proportion of drug expenditure, doctors increase the non-drug expenditure, rather than reducing the drug expenditure, of patients. Although this action does not alleviate the expenditure burden on patients, it distorts, to a certain extent, the expenditure structure of patients [1, 7]. Since doctors have information advantage and hospitals monopolize the medical services market, price regulation cannot reduce patients’ burden without changing the advantageous position of hospitals and doctors.

Since the implementation of the new healthcare reform in 2009, the government has been introducing several measures to reform the medical service market, such as encouraging the access of private hospitals and relaxing the price regulation on some hospitals [5, 24–26]. Some public hospitals restructured to private hospitals, and a large number of new private hospitals entered the medical market. Before 2013, the government had different pricing policies for these private hospitals: Whereas the pricing of medical services was unregulated for private for-profit hospitals, private nonprofit hospitals implemented government-guided pricing. That is, the pricing of private nonprofit hospitals, most of them reformed from public hospitals, was subject to price regulation policies, similar to that of public hospitals. In September 2013, the State Council of China issued a policy that explicitly required the deregulation of the pricing of medical services by nonpublic hospitals. The series of reforms implemented under the aforementioned policy ensures that both for-profit and nonprofit private hospitals in China abolish price regulation and perform independent pricing. Since the cancellation of the price regulation of private nonprofit hospitals by the government, all private hospitals have been implementing market pricing, whereas all public hospitals have been continuing to implement the government’s price regulation (as Table 1). This ensures a good policy environment to examine the impact of price regulation on hospital behavior and patients’ medical expenditure.

**Table 1:** Government’s price regulation policies for medical services in China

		Pricing before September 2013	Pricing after 2014
Government-run hospitals (public hospitals)		Government guided	Government guided
Private	Nonprofit	Government guided	Market independent
	For-profit	Market independent	Market independent

In the 1970s, some states in the United States implemented a fixed-rate price regulation approach for health care providers to limit the maximum pricing of health care services in order to alleviate the problem of rapidly rising health care expenditures [27, 28]. Some studies have concluded that rate regulation has been effective in controlling the growth of health care expenditures. Some theoretical analyses of the effects of price regulation are also available in China [8, 12], it offers limited evidence to support these inferences. This study attempts to overcome the aforementioned research gap and empirically examine the impact of medical services pricing deregulation on patients’ expenditure. For this purpose, we establish a DID model to compare the changes in inpatients’ medical expenditure and expenditure structure in private nonprofit hospitals before and after price deregulation, keeping public hospitals as the control group. Further, to clarify the influence of market competitiveness on hospital decision-making and perform robustness evaluation, we use data pertaining to the differences in competitiveness among hospitals.

The paper comprises four parts: In the first part, we discuss relevant data and variables, and, in the second, we describe the study’s setting and establish a DID model to test the impact of deregulation on patients’ medical expenditure. The third part presents the empirical study, for which we mainly use patient-level expenditure data to analyze the behaviors of over medical treatment. Finally, the fourth part depicts the implications of the study’s findings and presents the study’s conclusions.

## Methods

### Data sources

In this study, we used the claim data of insured inpatients residing in a major Chinese city for the period 2010–2015. The population of this city was over 16.3 million in 2018 (close to that of the Netherlands), and it ranked 8th in annual GDP among Chinese cities. The dataset was assembled by the National Health Commission and covered all hospitals in this city. The data included variables such as the expenditure for each inpatient visit, length of hospital stay, age, gender, and ICD code (International Classification of Diseases code). These data were the real-time recording of insured patients and accurately record the expenditure, occurrence time, and patient outcomes during the period of hospitalization. In the regression analysis performed in this study, we excluded the diseases for which the total number of patients is less than 500, to reduce the impact of rare diseases on regression results. Further, we mainly examined the changes in inpatient expenditure and expenditure structure before and after the implementation of price deregulation. The patients who were treated in private nonprofit secondary hospitals formed our experimental group, whereas those treated in public nonprofit secondary hospitals that were subject to price regulation before and after the policy change formed the control group. To avoid the impact of rank evaluation of hospitals, we excluded the hospitals that were rated as tertiary hospitals during the study period. Further, since some private hospitals were not appointed hospitals offering medical insurance, only 14 private nonprofit secondary hospitals were included in the data analysis. Moreover, two hospitals were excluded because they were rated as tertiary hospitals in 2015. Finally, the experimental group included 12 secondary hospitals and 40,411 patient samples. The total patient sample is 250,270.

### Variables

This study focused on the total and itemized expenditures of inpatients per hospital visit, such as diagnostic test, drug, and physician service expenditure. Further, we examined the expenditure structure, such as the proportions of diagnostic test expenditure and drug expenditure. To reduce statistical bias, we removed the samples whose total expenditure was missing or 0 (about 3000). Moreover, regarding patient characteristics, the study mainly considered age and gender. Gender was represented using a dummy variable, whose values were 1 for male patients and 0 for female patients. In addition, to ensure the comparability of expenditures, we controlled the diagnostic ICD code of inpatients, that is, the main diagnosis results of patients in a hospital. To exclude the influence of extreme values, we deleted the samples who were more than 80 years old or who stayed more than 200 days in a hospital.

### Models

The study establishes a DID econometric model to investigate the changes in patients’ medical expenditure after price deregulation:

1 $$ {Y}_{it}={\beta}_0+{\beta}_1\ast {T}_t+{\beta}_2\ast {D}_i+{\beta}_3\ast {T}_t\ast {D}_i+{\beta}_4\ast {X}_{it}+{\mu}_t+{\varepsilon}_{it} $$

Where *Y*_*it*_ represents inpatients’ total expenditure, diagnostic test expenditure, drug expenditure, physician service expenditure, the proportion of the diagnostic test expenditure, and the proportion of the drug expenditure. Further, all the expenditure data are expressed in the logarithmic form. In the equation, *T*_*t*_ time_t_ indicates the patient’s visit time. There will be a certain delay from the promulgation of policies to the implementation of policies, so we regarded the first half of 2014 as the time for local governments to implement policies. If the patient visited before September 2013, then *T*_*t*_ = 0 time_t_ = 0time = 0; If the patient visited after the second half of 2014, then *T*_*t*_ = 1 time_t_ = 1time = 1. Further, *D*_*i*_ treat_i_ refers to either the experimental or control group. If the patient that treated in a private nonprofit secondary hospital, then *D*_*i*_ = 1 treat_i_ = 1treat = 1; however, if the patient treated in a public nonprofit secondary hospital, then *D*_*i*_ = 0 treat_i_ = 0treat = 0. *X*_*it*_ is the control variable and mainly indicates the patient’s personal characteristics and ICD codes. To exclude the impact of hospitalization days on expenditure, we controlled for the number of hospitalization days of inpatients, as well. In addition, in clinical practice, the occurrence of complications significantly affect inpatient expenditure; hence, we controlled for the dummy variable to indicate the occurrence of complications during hospitalization. If complications occur during hospitalization, then *complication*_*it*_ = 1 complication = 1; however, if no complication occurs during hospitalization, then *complication*_*it*_ = 0 complication = 0. Finally, we controlled for the type of patients’ medical insurance, as well. *μ*_*t*_ is time fixed effect.

## Results

### Parallel trend assumption

**Fig. 1:**
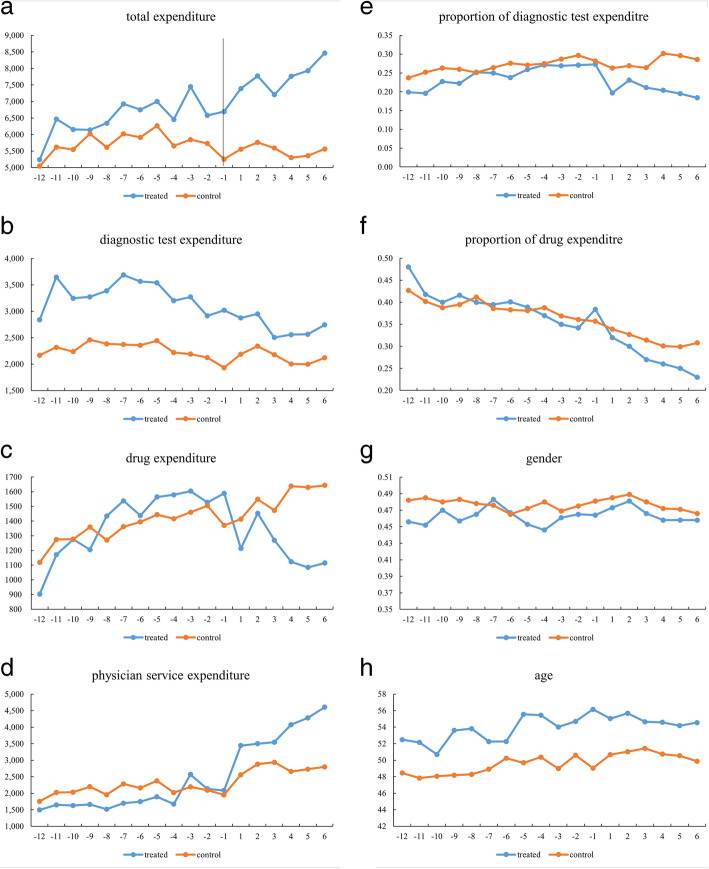
Parallel Trend. *Note*: On the horizontal axis, 1 represents the third quarter of 2014, − 1 represents the last quarter of 2013, and so on. (**a**-**h**) in figure 1 depict the mean values of inpatient total expenditure per inpatient visit, inpatient diagnostic test expenditure per inpatient visit, inpatient drug expenditure per inpatient visit, inpatient physician service expenditure per inpatient visit, the proportion of diagnostic tests expenditure per inpatient visit, the proportion of drug expenditure per inpatient visit, the gender of per inpatient visit, the age of per inpatient visit, respectively

At the core of DID identification strategy lies the so-called parallel trends assumption. A graphical representation of the parallel trend is provided in Fig. 1.

Figure 1 shows the stable difference or no difference between private nonprofit and public nonprofit secondary hospitals in all terms before price deregulation. However, descriptive statistics can be affected by extreme values, and we developed a difference model to further test the parallel trend hypothesis:

2 $$ {Y}_{it}={\beta}_0+{\beta}_1\ast {D}_i+{\beta}_2\ast {quarter}_t+{\beta}_3\ast {D}_i\ast i.{quarter}_t+{\beta}_4\ast {X}_{it}+{\mu}_t+{\varepsilon}_{it} $$

**Fig. 2:**
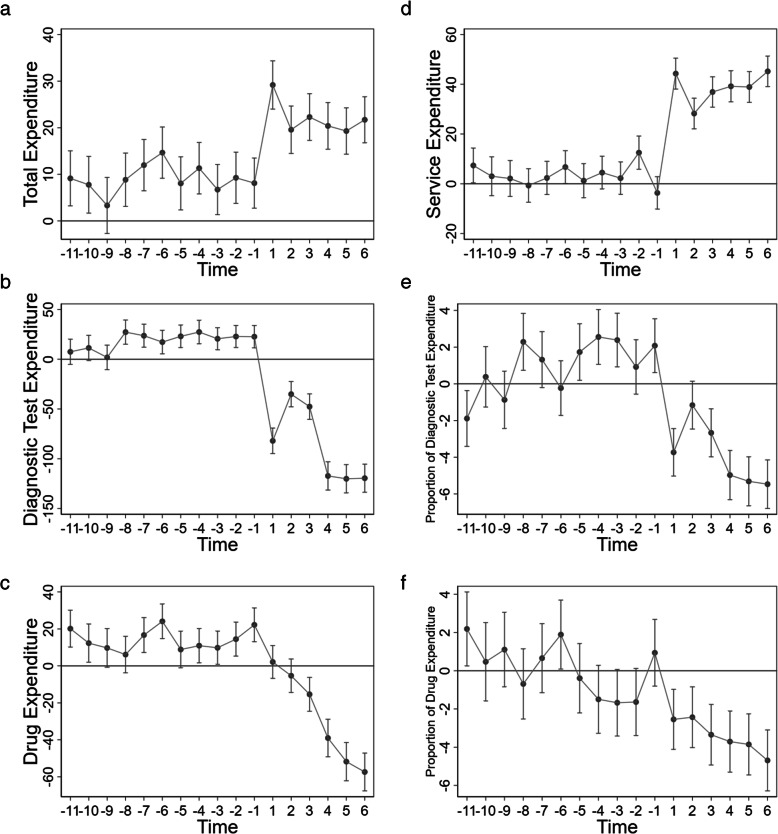
Parallel Trend Test. *Note*: The first quarter of 2010 is the control group. On the horizontal axis, 1 represents the third quarter of 2014, − 1 represents the last quarter of 2013, and so on. (**a**-**f**) in figure 2 depict the effects on inpatient total expenditure per inpatient visit, inpatient diagnostic test expenditure per inpatient visit, inpatient drug expenditure per inpatient visit, inpatient physician service expenditure per inpatient visit, the proportion of diagnostic tests expenditure per inpatient visit, and the proportion of drug expenditure per inpatient visit, respectively

Where all the variables are same as in Eq. 1, except the time variable *T*_*t*_. In Eq. 2, we make a preliminary regression of the complete sample on the quarterly time level to observe the trend of changes in inpatient expenditure and expenditure structure with time. The results are presented in Fig. 2, showing that there is no evidence to reject the parallel trends assumption.

Figure 2 depicts parallel trend between private nonprofit and public nonprofit secondary hospitals in terms of the total, diagnostic test, and drug expenditures of each inpatient visit before price deregulation; however, the differences between the two types of hospitals significantly changed after price deregulation. Before the deregulation, the total expenditure per inpatient visit of private nonprofit hospitals was slightly higher than that of public nonprofit hospitals. The average diagnostic test and drug expenditures per inpatient visit were higher, as well, whereas the physician service expenditure per inpatient visit shows no significant difference between the two hospital types. However, the differences between the two types of hospitals were stable, with a parallel trend. Following price deregulation, the difference in total expenditure per inpatient visit between private and public nonprofit hospitals significantly increased. Compared with the corresponding values of public nonprofit hospitals, the diagnostic test and drug expenditures per inpatient visit of private nonprofit hospitals significantly decreased, whereas the physician service expenditure per inpatient visit significantly increased after price deregulation.

In terms of expenditure structure, public and private nonprofit hospitals showed no significant difference before price deregulation. However, following the implementation of price deregulation, the proportions of diagnostic test expenditure and drug expenditure per inpatient visit of private nonprofit hospitals significantly decreased compared to that of public nonprofit hospitals.

### Impact of price deregulation on inpatients’medical expenditure

Table 2 reveals that compared with public nonprofit hospitals, private nonprofit hospitals that were deregulated significantly increased the total expenditure per inpatient visit by 10.5%, this corresponds to an increase of $672 in the total expenditure of per patient visit, after price deregulation. This evidence show that private nonprofit hospitals will increase relevant medical prices above the cost to ensure their profitable operation after price deregulation. Meanwhile, private nonprofit hospitals may achieve service premium through differential competition, that is, by improving their service quality and hospital environment and attracting more patients who are less sensitive to price but more interested in the hospital environment. Regarding itemized inpatient expenditure, compared with the relevant expenditure of public nonprofit hospitals, the diagnostic test and drug expenditure per inpatient visit of private nonprofit hospitals decreased significantly, whereas the physician service expenditure per inpatient visit increased significantly. In other words, to adapt themselves to market competition, private nonprofit hospitals pay more attention to the value of physicians’ service and improve their hospital medical service efficiency. For expenditure structure, the proportions of drug expenditure and diagnostic test expenditure per inpatient visit significantly decreased by 5.7 and 3.1%, respectively. This proves that private nonprofit hospitals will initiate the optimization of the structure of patients’ medical expenditure after price regulation.

**Table 2:** Results revealing the impact of price deregulation on patients’ medical expenditure

	Total expenditure	Diagnostic test expenditure	Drug expenditure	Service expenditure	Proportion of diagnostic test expenditure	Proportion of drug expenditure
Privite	0.059***	0.076***	0.245***	−0.117***	− 0.019***	0.064***
	(0.006)	(0.011)	(0.010)	(0.007)	(0.002)	(0.002)
Time	−0.107***	− 0.056***	− 0.441***	0.013*	0.045***	− 0.089***
	(0.007)	(0.013)	(0.010)	(0.008)	(0.001)	(0.001)
Privite#time	0.105***	−1.175***	−0.445***	0.343***	−0.057***	− 0.031***
	(0.008)	(0.024)	(0.016)	(0.010)	(0.002)	(0.002)
Gender	−0.005	−0.015*	− 0.013***	0.000	− 0.000	− 0.002***
	(0.003)	(0.008)	(0.005)	(0.004)	(0.001)	(0.001)
Age	0.017***	0.050***	0.015***	−0.002***	0.005***	0.000*
	(0.000)	(0.001)	(0.000)	(0.000)	(0.000)	(0.000)
Days_inpatient	0.036***	0.011***	0.041***	0.047***	−0.005***	0.001***
	(0.002)	(0.001)	(0.002)	(0.002)	(0.000)	(0.000)
Insurance	0.141***	0.140***	0.170***	0.028***	0.006***	0.024***
	(0.005)	(0.009)	(0.006)	(0.006)	(0.001)	(0.001)
Complication	0.175***	−0.324***	0.271***	0.197***	−0.030***	0.017***
	(0.006)	(0.013)	(0.009)	(0.007)	(0.001)	(0.001)
Constant	7.101***	4.807***	5.928***	6.459***	0.158***	0.345***
	(0.027)	(0.065)	(0.036)	(0.030)	(0.006)	(0.006)
ICD code	Yes	Yes	Yes	Yes	Yes	Yes
Time fixed effect	Yes	Yes	Yes	Yes	Yes	Yes
N	237,521	236,723	237,101	236,685	235,568	235,946
R-squared	0.357	0.155	0.335	0.378	0.243	0.322

The dependent variable for all expenditures is in logarithmic form. The estimates presented here were obtained from six difference-in-differences models, and regression coefficients are reported as elasticity and interactions as marginal effects. The significance levels are as follows: *p < 0.1 and ***p < 0.01. Finally, the numbers in parentheses represent robust standard errors

Regarding other control variables, although gender had no significant impact on total inpatient expenditure, the drug expenditure for men was slightly lower than that for women. Further, although the length of hospitalization has a significant positive effect on patients’ expenditure and a slight negative effect on the proportion of diagnostic test expenditure and drug expenditure. This may be because total expenditure will always increase, but diagnostic test expenditure will not always increase, with the length of hospitalization. Therefore, when the hospitalization and nursing expenses increase with the length of hospitalization, the proportion of diagnostic test expenditure will show a downward trend. Compared to patients with Basic Medical Insurance for Urban Residents, patients with Basic Medical Insurance for Urban Employees record significantly higher values for various expenditures and expenditure proportions.

### Heterogeneity analysis of market competitiveness

Public hospitals have always played a leading role in the development of China’s medical market. Due to strict barriers to entry, public hospitals dominate the medical services market and are the first care choice for residents. Contrarily, private hospitals have to develop their own comparative advantages, that is, develop treatment options to establish market competitiveness to obtain a small share of the medical market. We identify the competitive diseases in hospitals using two indicators (see Eqs. 3 and 4).

The hospital share of diseases refers to the proportion of the total number of patients with disease *a* admitted in hospital *A* among the total number of patients admitted in hospital *A* for 3 years, 2011–2013, and it directly reflects the importance of disease *a* in the medical service of *A* hospital. The higher the ratio, the stronger the capacity of the disease to affect patients, that is, become the dominant disease in the hospital. However, in the corresponding calculation process, this index does not efficiently represent the dominant diseases in hospitals, since some common diseases will have a large number of patients, as well. Therefore, we also used the market share index of diseases to define the dominant diseases of hospitals. The market share of diseases refers to the proportion of the total number of patients with disease *a* admitted in hospital *A* to the total number of patients with disease *a* admitted to the entire medical market over a period of 3 years. The higher the ratio, the more the hospital’s advantages in treating the disease. However, for some rare diseases, even if hospital *A* receives fewer patients each year, the index may retain a high value. Therefore, we combine the two aforementioned indicators to define a hospital’s dominant diseases, that is, when the hospital share of disease *a* is within the top 20% of the hospital and the market share of disease *a* is within the top 30% of the hospital, disease *a* is defined as a dominant disease of the hospital. This calculation of dominant diseases excludes the disadvantages of using the two indicators separately and, moreover, defines the hospital’s dominant diseases effectively.

3 $$ hosp\_ sh\mathrm{a} re\_a=\frac{the\ total\ number of\ patients\ with\ disease\ a\  admitted\ in\ hospital\ A}{the\ total\ number\ of\ patients\ admetted\ in\ hospital\ A} $$

4 $$ market\_ share\_a=\frac{the\ total\ number of\ patients\ with\ disease\ a\  admitted\ in\ hospital\ A}{the\ total\ number\ of\ patients\ with\ disease\ a\  admitted\ in\ the\ whole\ market} $$

Compared to the results depicted in Tables 2 and 3 reveals that the growth rate of total expenditure per inpatient visit of private nonprofit hospitals for dominant diseases is much higher than that of the full sample, and the rates of decline of diagnostic test expenditure and drug expenditure per inpatient visit are higher than the corresponding rate for the full sample, as well. Further, the physician service expenditure per inpatient visit, reflecting the service value of medical staff, is much higher than that of the full sample. From the perspective of expenditure structure, the proportion of diagnostic test expenditure and drug expenditure per inpatient visit for dominant diseases in private nonprofit hospitals significantly decreased after price regulation, and this decline was greater than the decline for the complete sample. For non-dominant diseases, Table 4 prove that the growth rate of total expenditure per inpatient visit for private nonprofit hospitals is lower than that of the full sample. Among itemized expenditure, the rate of decline of inpatient diagnostic test expenditure and drug expenditure per inpatient visit is lower than that of the full sample, whereas the increase rate of physician service expenditure per inpatient visit is lower than that of the full sample. Further, in terms of the expenditure structure, the rate of decline of the proportion of diagnostic test expenditure and drug expenditure per inpatient visit for non-dominant diseases is less than that of the full sample.

**Table 3:** Impact of price deregulation on patients’ medical expenditure for dominant diseases

	Total expenditure	Diagnostic test expenditure	Drug expenditure	Service expenditure	Proportion of diagnostic test expenditure	Proportion of drug expenditure
Privite	0.029***	0.135***	0.263***	−0.166***	0.005*	0.051***
	(0.010)	(0.021)	(0.019)	(0.012)	(0.003)	(0.003)
Time	−0.151***	−0.028	− 0.396***	− 0.050***	0.037***	− 0.073***
	(0.016)	(0.028)	(0.023)	(0.018)	(0.002)	(0.002)
Privite#time	0.136***	−1.524***	−0.857***	0.472***	−0.081***	− 0.051***
	(0.014)	(0.043)	(0.032)	(0.018)	(0.004)	(0.004)
Constant	7.670***	5.575***	6.665***	7.010***	0.138***	0.387***
	(0.074)	(0.079)	(0.080)	(0.089)	(0.007)	(0.007)
ICD code	Yes	Yes	Yes	Yes	Yes	Yes
Time fixed effect	Yes	Yes	Yes	Yes	Yes	Yes
Control variables	Yes	Yes	Yes	Yes	Yes	Yes
N	67,011	66,814	66,881	66,781	66,481	66,548
R-squared	0.281	0.164	0.278	0.302	0.284	0.253

The dependent variable for all expenditures is in logarithmic form. and we controlled for gender, age, length of hospitalization, insurance type, complication, ICD code, and time fixed effect too. Regression coefficients are reported as elasticity and interactions as marginal effects. The significance levels are as follows: *p < 0.1 and ***p < 0.01. The numbers in parentheses represent robust standard errors

**Table 4:** Impact of price deregulation on patients’ medical expenditure for non-dominant diseases

	Total expenditure	Diagnostic test expenditure	Drug expenditure	Service expenditure	Proportion of diagnostic test expenditure	Proportion of drug expenditure
Privite	0.068***	0.042***	0.231***	− 0.102***	−0.029***	0.069***
	(0.007)	(0.012)	(0.011)	(0.008)	(0.002)	(0.002)
Time	−0.092***	− 0.068***	− 0.459***	0.032***	0.049***	− 0.095***
	(0.007)	(0.014)	(0.011)	(0.008)	(0.002)	(0.001)
Privite#time	0.100***	−0.957***	− 0.191***	0.284***	− 0.046***	− 0.019***
	(0.009)	(0.029)	(0.016)	(0.012)	(0.002)	(0.002)
Constant	7.053***	4.709***	5.875***	6.362***	0.160***	0.353***
	(0.025)	(0.066)	(0.036)	(0.028)	(0.006)	(0.006)
ICD code	Yes	Yes	Yes	Yes	Yes	Yes
Time fixed effect	Yes	Yes	Yes	Yes	Yes	Yes
Control variables	Yes	Yes	Yes	Yes	Yes	Yes
N	170,510	169,909	170,220	169,904	169,087	169,398
R-squared,	0.382	0.148	0.373	0.404	0.227	0.350

The dependent variable for all expenditures is in logarithmic form. and we controlled for gender, age, length of hospitalization, insurance type, complication, ICD code, and time fixed effect too. Regression coefficients are reported as elasticity and interactions as marginal effects. The significance levels are as follows: ***p < 0.01. The numbers in parentheses represent robust standard errors

The aforementioned results reveal that price regulation constrains the optimal pricing behavior of hospitals. Once price regulation is implemented, hospitals lose their pricing independence and can only accept the government-guided price. However, the government-guided price is often lower than the cost of medical services and, in particular, excessively underestimates the value of medical personnel’s services. The financial subsidy implemented by the government is less than 10% of the revenue of public hospitals, and hospitals must constantly generate revenue by themselves, which causes distorted pricing in the medical service market. Following price deregulation, private nonprofit hospitals should provide medical services at market price and improve their pricing efficiency. However, in general, these hospitals do not completely follow the market’s pricing rules after deregulation. For dominant diseases, private hospitals change the traditional pricing model, increase the average inpatient total expenditure, adjust the expenditure structure, highlight the physician service value of hospital personnel, and reduce the diagnostic test and drug expenditures. To some extent, they correct distortions in the traditional pricing model and improve the efficiency of medical service pricing. However, private hospitals inevitably have to compete with public hospitals in pricing their medical services. With respect to non-dominant diseases, there is a direct competitive relationship between private and public hospitals, and private hospitals are at a disadvantage. Therefore, the private hospitals’ adjustment range of expenditure and expenditure structure of non-dominant diseases is relatively small and the pricing of these hospitals is close to that of public hospitals.

## Discussion and conclusions

After examining the pricing deregulation reform of private nonprofit hospitals in detail, we verified that if hospitals have independent pricing power, the inpatient expenditure and expenditure structure will change significantly. Under the policy experimental background of price deregulation, the estimation bias was effectively avoided by comparing private nonprofit and public nonprofit hospitals. The empirical results reveal that after price deregulation, hospitals will adjust the inpatients’ expenditure structure, improve medical service pricing, and reduce drug and diagnostic test expenditures. Further, most services of hospitals are priced significantly lower than their cost. Therefore, after price deregulation, private nonprofit hospitals increase the service price above the cost and realize their service premium by offering more physician services. Further, although these hospitals increase the average inpatient expenditure to a certain level, they optimize the expenditure structure. In this study, we defined the dominant diseases of private hospitals to verify the impact of market competitiveness on hospitalization expenditure and found that private hospitals significantly improve the physician service price of dominant diseases and adjust the expenditure structure after deregulation. This improves the hospitals’ pricing efficiency and their competitiveness in the medical market. For non-dominant diseases, private hospitals directly compete with public hospitals to price their services; hence, their pricing approach is similar to that of public hospitals.

Due to the implementation of pricing regulation, private medical institutions in China are unable to compete fairly with public medical institutions. Hence, the market entry of private hospitals does not significantly improve the degree of competition in the medical services market. Therefore, the market entry of private medical institutions cannot fundamentally solve the problem of insufficient supply in China’s medical service market. Further, the study’s findings indicate that by marketizing public hospitals and maintaining fair competition among public and private hospitals, the Chinese government can ensure citizens access to better medical services and reduce their medical burden.

## Data Availability

The datasets generated and/or analyzed during the current study are not publicly available due the data confidentiality agreement, but are available from the corresponding author on reasonable request.

## Abbreviations

DID: Difference-in-difference
FFS: Fee-for-service
ICD code: International Classification of Diseases code
